# Racial and Ethnic Heterogeneity in the Association Between Total Cholesterol and Pediatric Obesity

**DOI:** 10.3390/ijerph13010019

**Published:** 2015-12-23

**Authors:** Laurens Holmes, Alex LaHurd, Emily Wasson, Lavisha McClarin, Kirk Dabney

**Affiliations:** 1 Nemours/A.I. duPont Children’s Hospital, Nemours Office of Health Equity & Inclusion, 2200 Concord Pike, 8th Floor, Wilmington, DE 19803, USA; alahurd@udel.edu (A.L.); kdabney@nemours.org (K.D.); 2 Biological Sciences Department, University of Delaware, Newark, DE 19711, USA; 3 Biology Department, Gettysburg College, 300 North Washington Street, Gettysburg, PA 17325, USA; wassonemily326@gmail.com; 4 Epidemiology and Biostatistics Department, School of Public Health, University of Maryland-College Park, College Park, MD 20742, USA; lavisha.mcclarin@nemours.org

**Keywords:** racial heterogeneity, total cholesterol, pediatric, health disparities, obesity, BMI

## Abstract

Total cholesterol (TC) directly correlates with overweight/obesity, but it remains unclear if this association varies by race and ethnicity. We assessed the association as well as the racial/ethnic heterogeneity in this relationship. Data on 63,863 children were assessed using electronic medical records between 2010 and 2011. A cross-sectional design was utilized with log-binomial regression model and chi-squared statistic to examine the data. Overall, abnormal total cholesterol (ATC) was 7.5% (4812). Significant racial variability in ATC was observed: Black/African American (AA) (7.4%), White (7.0%), Asian (5.1%) and some other race (SOR) children (11.3%), χ^2^ (5) = 141.5, *p* < 0.0001. Black/AA (34.7%) and SOR children (41.2%) were predominantly overweight/obese, unlike the Asian children, (25.8%), χ^2^ (5) = 324.6, *p* < 0.0001. The BMI percentile was highest among SOR (69.0 ± 28.6) and Black/AA children (65.2 ± 29.1), but lowest among Asian children (55.7 ± 31.5). A significant racial variability was also observed in weight, with the highest mean among Black/AA children (36.8kg ± 23.0) and the lowest among Asian children (28.7kg ± 16.8), f = 7.2, *p* < 0.001. Relative to normal TC, children with ATC were 2.6 times as likely to have abnormal BMI, relative risk (RR) =2.60, 99% CI, 2.54–2.68). Compared to non-Hispanic (RR = 2.62, 99% CI, 2.54–2.69), the risk was lower among Hispanics (RR = 2.34, 99%, 2.21–2.48). Among children with ATC, risk for abnormal BMI was highest among Asians, adjusted RR = 2.91, 99% CI, 2.34–3.62), intermediate among AA (ARR = 2.68, 99% CI, 2.59–2.77), but lowest among Whites (ARR = 2.40, 99% CI, 2.39–2.64), and SOR (ARR = 2.33, 99% CI, 2.19–2.50). In a large sample of children, total cholesterol directly correlates with BMI, with an observed racial and ethnic heterogeneity.

## 1. Introduction

Serum lipids, namely triglycerides, total cholesterol (TC), high density lipoprotein (HDL) and low-density lipoprotein (LDL) levels have been shown to be associated with adiposity implying body fatness [1]. High levels of total cholesterol, triglycerides, and LDL have also been indicated to be directly associated with peripheral resistance [2,3,4,5] hypertension [6], and certain cardiovascular diseases such as arterial stenosis [5,7]. Epidemiologic data on serum lipids and the obesity epidemic have also identified a direct correlation between high total cholesterol and excessive body weight [8,9,10,11]. The link between blood lipids and body weight has been seen in adults [3,10] and children [12,13] in both clinical and population based studies. A direct correlation between serum lipids and skeletal muscle adiposity has also been observed [14,15,16]. Additionally, studies evaluating BMI and bone density have indicated some variability, while studies focusing on exercise and serum lipid correlation have indicated a reduction in serum lipid following sustained exercise [17,18]. Consequently, understanding the correlation between total cholesterol, adiposity, and body weight is essential in exploring the biomarkers of overweight/obesity among children. BMI as an age related marker of obesity of overweight has been previously shown requiring such adjustment in the process of understanding the role that BMI may possibly play in the overall understanding of total body fatness and obesity [19].

In pediatric studies, BMI percentiles are commonly used to examine childhood overweight/obesity [20]. These studies also address variability based on race and sex among children in the US, which has been recognized in higher BMI measures [21]. The determinants of this racial/ethnic and sex variability has not been fully understood.

Although total cholesterol has been utilized as a biomarker of overweight/obesity [14,15,18,19], it has not been characterized or stratified by race/ethnicity to account for the variance in the obesity epidemic among United States children. The current study provides a novel approach to bio-indicator characterization and stratification at the population level. Thus, data on racial/ethnic characterization and stratification of total cholesterol levels may provide some insight into the subpopulation predisposition to overweight/obesity. We aimed to assess racial/ethnic differences in total cholesterol among children, correlate this with BMI, and determine whether or not the differences in abnormal total cholesterol may explain in part the racial/ethnic variances in overweight/obesity among children.

## 2. Experimental Section

### 2.1. Materials and Methods

Following an Institutional Review Board approval, we conducted a cross-sectional study to examine the distribution of total cholesterol level by race/ethnicity to determine whether or not total cholesterol is associated with BMI differently across racial/ethnic groups. This design allows for a reliable and valid inference if information bias is addressed prior to data processing and analysis.

A cross-sectional design was used to assess preexisting data from the Nemours Children’s Health System electronic medical records (EMR) on the variables of interest, including race/ethnicity, total cholesterol level profile, body mass index (BMI), height, weight and type of medical insurance. The study population comprised 63,863 children seen at our healthcare system during 2010 and 2011.

#### 2.1.1. Sampling, Sample Size, and Power Estimation

A consecutive sampling technique involving the assessment of the EMR of all eligible patients was used. The eligibility criteria included patients with information on total cholesterol, insurance, zip code, BMI, race, and ethnicity. This method is adequate in examining inferential association in the data, given the representative nature of the sample.

To determine the power, implying the ability of the study to detect the minimal difference in total cholesterol levels between racial/ethnic groups, we used Type 1 error tolerance of 5% (0.05), effect size of 10%, and log binomial regression model, including margin probability, as an analytic tool. With these parameters, we estimated the power of the study to be adequate in assessing the difference in total cholesterol between Hispanic/Latino and non-Hispanic/Latino Blacks/AAs in comparison to Whites, as well as some other race (SOR) and Asians.

#### 2.1.2. Variable Ascertainment

The independent variable was total cholesterol as serum lipid profile. Using the American Academy of Pediatrics (AAP) guidelines on children and adolescents total cholesterol ascertainment, we categorized the total cholesterol into two categories: (a) accepted level and (b) high level. The accepted level, according to AAP is, ≤200 mg per 100 mL of blood whereas the high level is ≥200 mg per 100 mL of blood [22,23]. For the purpose of this study, we used normal and abnormal to represent the Academy’s acceptable and high levels of TC respectively. In order to determine how total cholesterol is associated wih obesity, we used the BMI as our outcome variable. The BMI measure was estimated based on the CDC guidelines [24] and was collected as a continuous variable using BMI percentages, BMI as a continuous variable and BMI as a dichotomous variable into normal *versus* higher BMI categories.

Race and ethnicity was used to account for the differences in BMI, as well as to assess how total cholesterol explains the racial/ethnic differences in BMI. Race which was self-reported was categorized as White, Black/AA, Asian, American Indian/Alaska Native, Hawaii Native/Pacific Islander and some other race (SOR). In this sample, some other race represented primarily Hispanics with an estimated 53% within this category. In order to determine whether or not other variables played confounding roles on how total cholesterol predicts BMI by race/ethnicity, we used type of medical insurance as a socioeconomic status proxy, since insurance has been shown to vary by race/ethnicity and socioeconomic factors have been linked to insurance, race and overweight. The insurance type was collected as a categorical variable and included private/commercial insurance, public insurance and uninsured (out of pocket payment for service). Weight and height were also collected as continuous variables with weight measured in kilograms and height in centimeters according to CDC guidelines [24].

### 2.2. Statistical Analysis

We first examined data for missing variables using frequency and percentages. For the race and ethnicity variables, we excluded from the summary statistics “information not available” as well as “refused to answer”. This exclusion accounted for less than 1% of the total sample implying a marginalized influence an acceptable in terms of data processing prior to model estimation. Additionally, we assessed the distribution and spread of the data. For the continuous variables, we preformed normality tests to determine the shape and spread of the data mainly for BMI.

To test the hypothesis on racial/ethnic differences in BMI, we used the Pearson chi squared statistic, and where necessary Fisher’s exact test to compensate for expected small cell count. Prior to the assessment of the relationship between total cholesterol and BMI, and the racial/ethnic heterogeneity therein, we assessed for race/ethnicity as effect measure modifier using Mantel-Haenszel stratification analysis. This analysis allowed for the race-specific stratum data as well as adjustment for race in the relationship between total cholesterol and BMI. Because we observed race and ethnicity as effect measure modifier, we built a multivariable model adjusting for specific race, hence racial/ethnic heterogeneity. The log binomial regression model was used to assess how total cholesterol predicts BMI. To determine the racial and ethnic differences on the association between TC and BMI, we used both adjusted risk ratio and risk difference from log binomial regression model to present the data.

The significance level was set at 5% (type 1 error tolerance) for univariable analysis that involved the chi squared test. For the univariable and multivariable log binominal regression model, the significance level was set at 1% (type 1 error tolerance) with margin probability. All analyses were two-tailed, and STATA statistical software (version 13.0) was used for the entire analysis (STATA Corporation, College Station, TX, USA).

## 3. Results and Discussion

### 3.1. Results

#### 3.1.1. Study Sample Characteristics

Table 1 demonstrates the overall study characteristics (non-stratified) and describes the distribution of ethnicity, race, BMI, total cholesterol, and insurance. Of the 63,863 participants in this study, 4812 (7.5%) had an abnormal total cholesterol. In terms of ethnicity, non-Hispanic/Latino comprised 89.4% of our sample while Hispanic/Latino comprised 10.6% of our sample. An estimated 33.4% of the children were overweight or obese (abnormal BMI). Abnormal total cholesterol was observed in 7.5% of the sample. Additionally, 47.5% of the children had public insurance in the form of Medicaid.

**Table 1:** Study sample characteristics.

Variable	Number	Percentage (%)
**Ethnicity**		
Hispanic/Latino	6803	10.6
Non-Hispanic/Latino	57,060	89.4
**Race**		
AI/AN	102	0.2
Asian	1324	2.2
Black/AA	31,485	49.5
HN/PI	43	0.07
SOR	5703	9.0
White	24,971	39.3
**Body Mass Index**		
Normal	42,564	66.6
Overweight/Obese	21,309	33.4
**Total Cholesterol**		
Normal < 200 mg	59,061	92.5
Abnormal ≥ 200 mg	4812	7.5
**Insurance**		
Commercial	31,550	49.4
Public	30,333	47.5
Uninsured	1990	3.1

*Notes and Abbreviations*: AA: African American, SOR: Some Other Race, HN/PI: Hawaiian Native/Pacific Islander, I/AN: American Indian/Alaska Native. Total cholesterol was ascertained using acceptable level as normal while high level as abnormal (American Academy of Pediatrics) The BMI was ascertained using the CDC guidelines for children BMI.

#### 3.1.2. Study Characteristics by Race/Ethnicity (Categorical Variables)

Table 2 presents study characteristics including total cholesterol distribution by race/ethnicity. The abnormal total cholesterol was highest among Black/AA children (7.4%) and SOR children (11.3%), intermediate among White children (7.0%) and the lowest among Asian children (5.1%, χ^2^ (7) = 141.9, *p* < 0.0001). Black/AA children (34.3%) and SOR children (40.6%) were observed to be predominantly overweight/obese while White children (30.0%) were intermediate. In contrast to SOR and Black/AA children, abnormal BMI was lowest among Asian children, 25.7%, χ^2^ (6) = 324.6, *p* < 0.0001. Significant variability was observed in the distribution of insurance where Black/AA children (61.4%) and SOR children (60.1%) were observed to be more likely associated with public insurance. White children (69.8%) and Asian children (64.5%) were more likely to be primarily associated with commercial insurance. Finally, Black/AA children (3.2%) and SOR children (3.5%) were more likely to be uninsured as well.

**Table 2:** Study characteristics by race (categorical variables).

Variable	Race							
	AI/AN	Asian	Black/AA	HN/PI	SOR	White	χ ^2^ (df)	*p*
	n(%)	n(%)	n(%)	n(%)	n(%)	n(%)		
**Ethnicity**							3,0004 (18)	<0.0001
Hispanic/Latino	12 (11.6)	21 (1.57)	434 (1.36)	20 (40.8)	3934 (68.2)	2357 (9.33)		
Non-Hispanic/Latino	90 (87.4)	1303 (97.7)	31,051 (97.3)	23 (46.9)	1769 (30.7)	22,614 (89.5)		
Refused	0 (0)	2 ( 0.15)	26 (0.08)	0 (0)	6 (0.10)	25 (0.10)		
Information NA	1 (0.97)	8 (0.60)	384 (1.20)	6 (12.2)	60 (1.04)	280 (1.11)		
**Body Mass Index**							321.3 (6)	<0.0001
Normal	71 (68.9)	999 (74.9)	20,959 (65.7)	35 (71.4)	3429 (59.4)	17,697 (70.0)		
Overweight/Obese	32 (25.1)	335 (25.1)	10,936 (34.3)	14 (28.6)	2340 (40.6)	7579 (30.0)		
**Total cholesterol**							141.9 (6)	<0.0001
Normal	96 (93.2)	1266 (94.9)	29,548 (92.6)	48 (97.0)	5116 (88.7)	23,493 (92.9)		
Abnormal	7 (6.80)	68 (5.10)	2347 (7.36)	1 (2.04)	653 (11.3)	1783 (7.05)		
**Insurance**							7400 (12)	<0.0001
Commercial	62 (60.2)	860 (64.5)	11,280 (35.4)	17 (34.7)	2104 (36.5)	17,635 (69.8)		
Public	39 (37.9)	435 (32.6)	19,586 (61.4)	31 (63.3)	3465 (60.10)	6915 (27.4)		
Uninsured	2 (1.90)	39 (2.9)	1029 (3.2)	1 (2.0)	200 (3.5)	726 (2.9)		

*Notes and Abbreviations*: Significant *p*-value ≤ 0.001; df: degree of freedom. AA: African American, SOR: Some Other Race, HN/PI: Hawaiian Native/Pacific Islander, AI/AN: American Indian/Alaska Native, NA: Not Available.

#### 3.1.3. Study Characteristics by Race (Categorical Variables)

**Figure 1:**
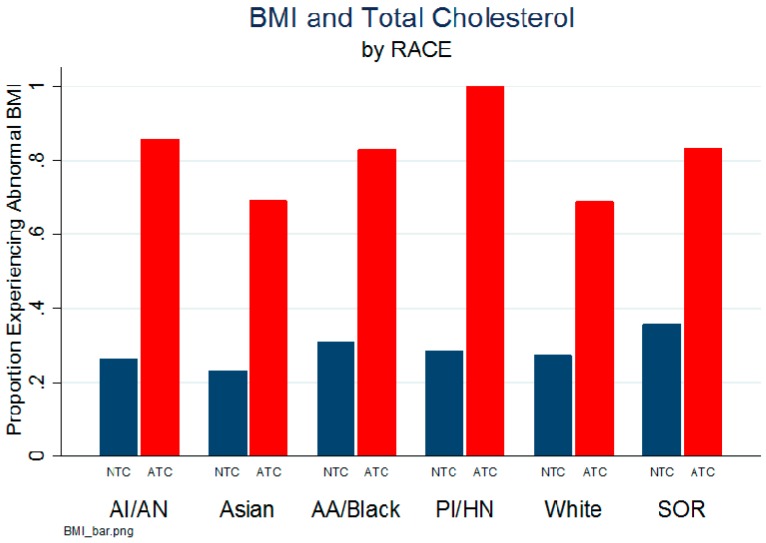
BMI and total cholesterol by race. NTC: Normal total cholesterol, ATC: abnormal total cholesterol.

Table 3 demonstrates the distribution of anthropomorphic variables namely height, weight, BMI percentile and BMI. The BMI percentile was highest among SOR children (69.0 ± 28.6) and Black/AA children (65.2 ± 29.1), intermediate among White children (62.4 ± 29.0) and lowest among Asian children (55.7 ± 31.5). A significant racial variability was observed by weight, with the mean weight highest among Black/AA children (36.8 kg ± 23.0), intermediate among SOR children (32.6 kg ± 20.5) and White children (34.0 kg ± 20.8), and lowest among Asian children (28.7 kg ± 16.8, f = 7.2, *p* < 0.001). The proportion of abnormal BMI, given total cholesterol (TC) is illustrated by race (Figure 1).

**Table 3:** Study characteristics by race (continuous variables).

	AI/AN	Asian	Black/AA	HN/PI	SOR	White	F	*p*
**Variables**	mean (SD)	mean (SD)	mean (SD)	mean (SD)	mean (SD)	mean (SD)		
Height	126.6 (25.6)	122.2 (25.0)	130.4 (27.3)	115.6 (25.6)	123.7 (25.6)	128.2 (26.9)	73.9	<0.0001
Weight	31.4 ((17.6)	28.7 (16.8)	36.8 (23.0)	26.5 (16.5)	32.6 (20.5)	34.0 (20.8)	71.2	<0.0001
**BMI%**	58.1 (33.1)	55.7 (31.5)	65.2 (29.1)	65.8 (26.4)	69.0 (28.6)	62.4 (29.0)	58.6	<0.0001
BMI	17.9 (3.79)	17.7 (3.69)	19.5 (13.1)	18.0 (3.36)	19.2 (4.85)	18.8 (11.2)	10.5	<0.0001

*Notes and Abbreviations*: Height and weight measured according to CDC guidelines for BMI [23]; Significant *p*-value ≤ 0.001. AA: African American, SOR: Some Other Race, HN/PI: Hawaiian Native/Pacific Islander, AI/AN: American Indian/Alaska Native, BMI: Body Mass Index, BMI%: Body Mass Index Percentage.

#### 3.1.4. Multi-Level Risk Ratio (adjusted) Relationship between Total Cholesterol and Other Factors with BMI

Table 4 presents the race stratified or race-adjusted model for the relationship between total cholesterol and BMI. Although not shown on the table, relative to normal total cholesterol, children with abnormal total cholesterol were 2.6 times as likely to have abnormal BMI (overweight/obesity) (ARR = 2.60, 99% CI, 2.55–2.65). There were significant differences in the stratum specific OR for the relationship between BMI and ATC, implying race as effect measure modifier. The adjusted OR = 8.93, 99% CI, 7.65–8.83.

**Figure 2:**
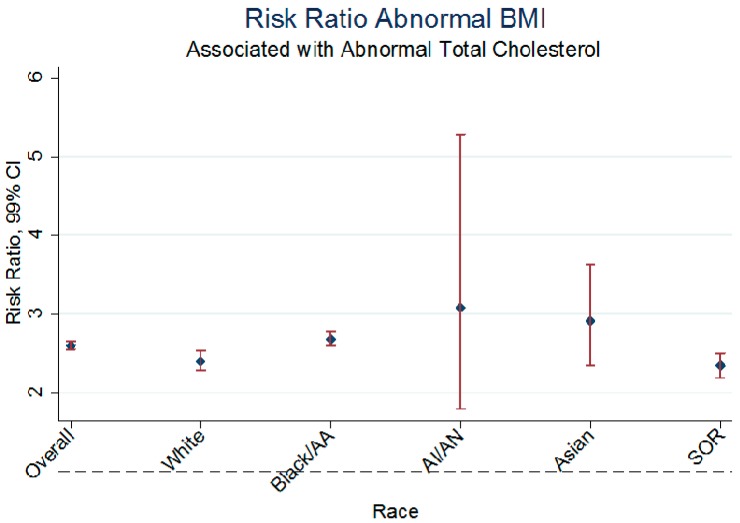
The overall and adjusted RR on the association between BMI and Total Cholesterol in Children, AA: African American; AI: American Indian; AN: Alaska Native; SOR: Some Other Race.

Table 5 presents the race-stratified relationship between TC and BMI, adjusted for insurance, sex and age. There remains racial and ethnic heterogeneity in the relationship between BMI and TC after adjustment for the potential confounders namely age, sex and insurance as the surrogate for access and care utilization (Figure 2). The risk for abnormal BMI given TC was highest among Asians and AI/AN, intermediate among Blacks/AA and lowest among Whites and SOR.

**Table 4:** The odds ratio effect modification by race on the association between total cholesterol and BMI in children.

Race	Odds Ratio	99% CI	*p*
AI/AN	16.8	1.68–168.0	0.001
Asian	7.3	4.24–12.64	<0.001
Black/AA	11.0	9.75–12–64	<0.001
White	5.8	5.23–6.49	<0.001
SOR	8.9	7.13–11.19	<0.001

*Notes and Abbreviations*: The Type 1 error level was 0.01 (1%), CI: Confidence Interval, AA: African American, SOR: Some Other Race, AI/AN: American Indian/Alaska Native.

**Table 5:** Adjusted multi-level risk ratio on the relationship between total cholesterol and BMI by race/ethnicity.

Variable	ARR	99% CI	*p*
Ethnicity			
Non-Hispanic	2.61	2.54–2.69	<0.001
Hispanic	2.34	2.21–2.48	<0.001
Race			
White	2.40	2.29–2.53	<0.001
Black/AA	2.68	2.60–2.77	<0.001
AI/AN	3.08	1.80–5.28	<0.001
Asian	2.91	2.34–3.62	<0.001
SOR	2.34	2.19–2.50	<0.001

*Notes and Abbreviations*: The Type 1 error level was 0.01 (1%), Significant *p*-value ≤ 0.001. RR: Risk Ratio, CI: Confidence Interval, AA: African American, SOR: Some Other Race, AI/AN: American Indian/Alaska Native, BMI: Body Mass Index, BMI%: Body Mass Index Percentage Adjusted for insurance, sex and age.

### 3.2. Discussion

The purpose of this study was to determine whether or not racial/ethnic heterogeneity exists in the relationship between total cholesterol and pediatric BMI. Studies in adult populations have previously shown the correlation between total cholesterol and total body fat as well as obesity. We postulated that total cholesterol may be distributed unequally across race and ethnic groups, which may explain in part the racial/ethnic variability in pediatric obesity. There are relevant findings based on our data and some of which are very novel. First, among children in our sample, there is a direct correlation between total cholesterol and BMI. Secondly, total cholesterol varies by race and ethnicity. Thirdly, our data confirm the relationship between total cholesterol and obesity, while the relationship between total cholesterol and BMI varies by race and ethnicity. These data affirm previously published studies on the direct correlation between total cholesterol and obesity [3,10,12,13,25].

Studies have shown in both adult [3,10] and children populations [12,13] as well as in animal models that lipids and triglycerides exposure are associated with weight gain and overweight/obesity. The body mass index (BMI) is a commonly used measure of excess body weight, but it is not a direct measure of adiposity [14,15]. Excessive body weight may reflect high bone density [16], which directly correlates with an increase in BMI [17]. Despite these findings, BMI remains a proxy in research for body weight. More specifically, it has been directly associated with blood lipids [18,19]. While total cholesterol and weight correlation have been fairly well discussed in the medical and health literature, very few data exist on the variability of this relationship by race and ethnicity, and indeed data are more sparse with respect to the pediatric population. We assessed the distribution of total cholesterol by race and ethnicity and found a substantial variability, with Black/AA and SOR more likely to have abnormal TC. We also observed that Asians had the lowest abnormal total cholesterol prevalence. These findings are plausible in the sense that the dietary profiles of Asians in the US tend to consist mainly of vegetables, fruits and white meat, as well as several antioxidant-related nutrients [26]. While we are unaware of data on the dietary profile of Asian children, it is imaginable that children of Asian origin in America are more likely to comply with the parental/adult dietary choices implying the ingestion of white meat, vegetables and fruits. In contrast Black/AA and SOR are largely associated with unhealthy diets that have high fat concentration, high sugar, and low vegetables [26,27]. Although dietary profiles remain a good indicator for total cholesterol, genetic factors do play a role in the bio-availability as well as metabolism of lipids, which may also vary by race and ethnicity. Unfortunately, we do not have any data to illustrate difference in the dietary profile or genetic factors of the racial and ethnic group in our sample. Availability of these data could have facilitated greatly the implication of total cholesterol in obesity, as well as its race/ethnicity variability. While total cholesterol contributes to an estimated 5.3% to potential risk markers for obesity, as per our data, it may account in part for the racial/ethnicity variability in the observed pediatric obesity epidemic where Black/AA females and Hispanic/Latino males tend to disproportionately bear the burden of this epidemic [12,21].

Our stratified analysis for assessing race as effect measure modifier indicated significant racial and ethnic differences in the association between BMI and total cholesterol. Abnormal total cholesterol in Asians was associated with a relatively increased weight gain. In contrast, SOR and Black/AA were less likely to demonstrate sensitivity to overweight given abnormal total cholesterol. It is unclear why we observed this variability and there are no data available to neither support nor refute our findings. However, we speculate that Black/AA, SOR and perhaps Whites have developed tolerance to triglycerides, lipids, and fatty diet to the extent that increases in these substrates provide a small increase in total body fat as well as body weight. We question the relevance of these findings to intervention.

In spite of the novelty of this study, namely variability in the total cholesterol sensitivity to overweight/obesity by race and ethnicity, there are some limitations. First, we utilized retrospective data and cross-sectional design which have tendencies for information bias as well as limiting temporal sequence with respect to cause and effect. Because we examined our data for reliability, as well as monitored the timing of serum collection and data on BMI from our EMR, it is not possible that our entire findings are driven by limited temporality as well as information biases. Secondly regarding the statistical stability of the findings on AI/AN as well as PI/HN, our subpopulation analysis was underpowered, implying emphasis on the point estimate and not on precision (*p* value) in the interpretation and application of these results. Thirdly, there are confounding to this relationship that we were unable to assess and address given the nature of pre-existing data. It is possible that unmeasured confounding might influence the result in this study. Finally, irrespective of the statistical software used for adjustment of confounding, residual confounding exist which may influence the results in this study [28].

## 4. Conclusions

In summary, there is a correlation between total cholesterol and obesity among children. Additionally, this relationship varies by race and ethnicity which may explain in part the racial/ethnic variability in childhood obesity epidemic in our nation. Despite the magnitude of these findings, we caution the interpretation as well as the application of these data due to its cross-sectional design and causal inference restriction, which does not allow us to clearly determine the directionality of TC on the causal pathway of childhood obesity as BMI.
